# Geographic expansion, not viral intensification, leads to human dengue outbreaks in Mexico: a 40-year integrated remote sensing and machine learning analysis

**DOI:** 10.3389/fpubh.2026.1888753

**Published:** 2026-08-03

**Authors:** Huixuan Li, Christopher Lee, Sean Sweeney, Homayoun Valafar, Melissa S. Nolan

**Affiliations:** 1 Department of Epidemiology and Biostatistics, University of South Carolina, Columbia, SC, United States; 2 Department of Computer Science and Engineering, University of South Carolina, Columbia, SC, United States; 3 Institute for Infectious Disease Translational Research, University of South Carolina, Columbia, SC, United States; 4 Artificial Intelligence Institute, University of South Carolina, Columbia, SC, United States

**Keywords:** dengue fever, EM-DAT, hazard covariates, machine learning, Mexico, spatiotemporal analysis

## Abstract

**Background:**

Dengue fever poses a pervasive, yet escalating public health burden in Mexico and abroad.

**Methods:**

We conducted a 41-year spatiotemporal analysis of dengue fever across Mexico (1985–2025), integrating monthly case surveillance with climate, land cover, vegetation, and novel disaster severity covariates derived from the Emergency Events Database. Four supervised regression models were trained on 1990–2021 data, with models evaluated on a 2022–2023 temporal holdout and against observed 2024–2025 surveillance totals. Five supplementary hazard analyses examined temporal correlation, disaster type breakdown, spatial co-occurrence, pre/post event trajectories, and sensitivity to scoring weight assumptions.

**Results:**

Mann–Kendall trend analysis identified statistically significant increasing dengue incidence in 15 of 32 states (9 inland), evidencing geographic expansion over four decades. Z-score analysis confirmed 2024 as a profound anomaly across both endemic and emerging states. Hazard features were significantly associated with national monthly dengue counts at lags 0–2 months across the full 1985–2025 series. A + 613% case increase following the June 2024 tropical storm. Sensitivity analyses confirmed the project’s developed ‘Severity Score’ performed comparably to four theoretically motivated differential weighting schemes.

**Conclusion:**

This study provides the first structured integration of disaster severity covariates in a dengue forecasting framework for Mexico. Geographic expansion into inland states, the 41-year trend analysis, and the hazard adjustment results collectively support a differentiated public health response that targets both endemic coastal states and newly affected inland regions. The analytical framework is directly transferable to emerging dengue risk contexts in the United States and Central America, geographic neighbors also experiencing increased dengue virus transmission.

## 1 Introduction

Dengue fever is the most prevalent mosquito-borne arboviral disease worldwide, with an estimated 390 million infections occurring annually across more than 129 countries (1). The global burden has tripled over the past three decades, driven by urbanization, climate change, international travel, and the geographic expansion of the primary vector, *Aedes aegypti* (2–4). Latin America and the Caribbean bear a disproportionate share of this burden, recording 12.6 million dengue cases in 2024 alone, the highest annual total in the region’s surveillance history (5).

Mexico is a hyperendemic country situated at the intersection of multiple dengue risk factors: a vast tropical and subtropical coastal zone, active hurricane corridors on both the Pacific and Gulf coasts, rapid urbanization that creates ideal *Aedes* breeding habitats, and a population of approximately 130 million with heterogeneous immunity across the four circulating serotypes (DENV-1 through DENV-4). Over 883,000 confirmed dengue cases were recorded in Mexico’s 41-year integrated surveillance dataset, with the 2024 epidemic of 127,741 cases representing the largest annual burden on record (6), a 135% increase over 2023 and 2.35 times the next-highest year in the series. The state-level concentration of transmission is pronounced: Veracruz, Jalisco, Guerrero, Sinaloa, and Nuevo León together accounted for approximately 41% of cumulative cases from 1985 to 2025. At the same time, a pattern of geographic expansion into historically unaffected inland states has been documented, raising concern about the capacity of public health systems in these regions to detect and respond to dengue outbreaks.

Despite this burden, Mexico lacks the kind of high-resolution, spatially explicit dengue forecasting framework that has been developed for other hyperendemic settings, such as Brazil, Thailand, and Singapore. Published forecasting approaches for Mexico have largely relied on national-level time series decomposition (7), weekly cluster-level regression (8), or ecological correlational analyses (9), without prospective validation against recent epidemic years or integration of multi-source environmental covariates at the state-month resolution. A critical gap is the absence of structured disaster event covariates, despite decades of evidence linking storms, floods, and extreme temperatures to subsequent dengue amplification through mosquito breeding site proliferation, population displacement, and healthcare system disruption (10–12).

Climate-driven changes in vector ecology have been extensively modeled (13–16), and the global distribution of dengue risk has been mapped at high resolution (17–19). However, the integration of disaster event data as a structured, time-varying covariate in dengue forecasting models represents a largely unexplored methodological direction, and the Emergency Events Database (EM-DAT) maintained by the Centre for Research on the Epidemiology of Disasters offers a uniquely comprehensive historical record of disaster events with the temporal and geographic specificity required for monthly state-level modeling. Supervised machine learning methods, particularly ensemble tree-based approaches, have shown strong performance in dengue forecasting tasks by capturing nonlinear relationships between environmental covariates and disease incidence without pre-specifying functional forms (20–22). Random Forest and XGBoost have emerged as leading algorithms for structured tabular environmental health data, offering both predictive performance and post-hoc interpretability through feature importance and (SHapley Additive exPlanations) SHAP analysis (23, 24).

This study addresses current literature gaps by developing and validating a state-level monthly dengue forecasting framework for Mexico spanning 1985–2025, with the following specific objectives: (1) characterize long-term spatiotemporal trends in dengue incidence across all 32 Mexican states, including identification of states experiencing geographic emergence; (2) develop and compare baseline and hazard-adjusted machine learning forecasting models, evaluated on a temporal holdout (2022–2023) and tested against observed 2024–2025 surveillance ground truth; (3) construct a novel EM-DAT-derived disaster severity covariate and validate its epidemiological coherence through five independent analyses; and (4) generate georeferenced outputs for ArcGIS software-based integration supporting spatial analysis, emerging hotspot detection, and risk communication.

## 2 Methods

### 2.1 Study area and time period

This study encompassed all 32 federal entities of Mexico (31 states and Mexico City), analyzed at the state level on a monthly temporal resolution spanning January 1985 through December 2025. The resulting panel dataset comprised 15,744 state–month observations across 41 years. This temporal scope was selected to capture multiple complete epidemic cycles, document the transition from early to hyperendemic dengue transmission, and include the record-breaking 2024 epidemic and the 2025 decline as contemporaneous out-of-sample test periods.

### 2.2 Human dengue surveillance data sources and processing

We assembled a monthly state level (ADM1) panel of Mexico spanning 1985 through 2025 (12 months x 32 states x 41 years). Dengue case counts were compiled from the weekly Boletín Epidemiológico bulletins published by Mexico’s Sistema Nacional de Vigilancia Epidemiológica (SINAVE/Dirección General de Epidemiología/Secretaría de Salud) and aggregated to monthly counts via ISO 8601 week to month mapping. State level population was derived from the Censos de Población y Vivienda published by Mexico’s Instituto Nacional de Estadística y Geografía (INEGI) for 1980, 1990, 1995, 2000, 2005, 2010, and 2020; intercensal years were linearly interpolated, post-2020 years linearly extrapolated, and monthly values obtained by upsampling.

Case counts were expressed as both absolute numbers and age-standardized incidence rates per 100,000 population using World Bank and CONAPO intercensal population projections denominated annually by state. Annual dengue age-standardized incidence rate per 100,000 population, per state was calculated using equation one listed in Supplementary file 1.

### 2.3 Environmental and climate covariate data sources and processing

**Figure 1:**
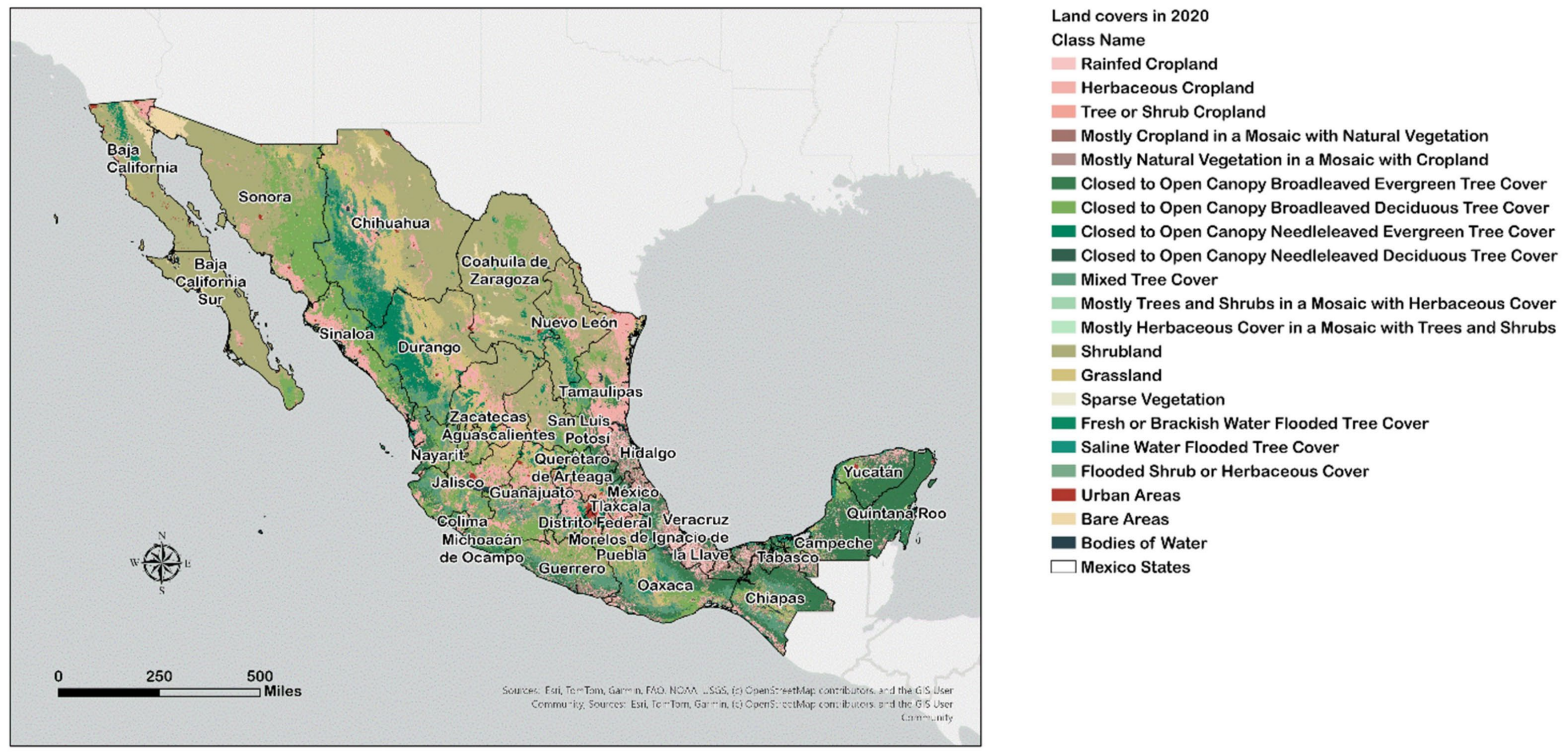
Study area and land cover classification across the 32 Mexican states^₸^.*^₸^* Land cover classification map for year 2020 shown as graphical example.

Land cover categories derived from the Copernicus Global Land Cover product, aggregated to state boundaries from the INEGI Marco Geoestadístico. Twenty-one land cover classes were represented, ranging from agricultural land uses (rainfed cropland, herbaceous cropland, tree or shrub cropland, and cropland-natural vegetation mosaics) to natural vegetation types (broadleaved and needle leaved tree cover, mixed tree cover, shrubland, grassland, and sparse vegetation), wetland and flooded environments (fresh or brackish water flooded tree cover, saline water flooded tree cover, flooded shrub or herbaceous cover), and anthropogenic and bare surfaces (urban areas, bare areas, bodies of water; Figure 1). Dominant land cover in the northern states is shrubland and sparse vegetation, transitioning to broadleaved evergreen and deciduous tree cover in the humid tropical south and southeast. Agricultural mosaics predominate in the central highlands. Land cover classification maps were produced using ArcGIS Pro v2.6.2 (Esri Corporation, Redwoods, CA).

Four remote sensing climate and vegetation indicators Agricultural Stress Index (ASI), Vegetation Health Index (VHI), Normalized Difference Vegetation Index (NDVI), and estimated precipitation were obtained from the Food and Agriculture Organization Agricultural Stress Index System (FAO-ASIS) at decadal cadence pre-aggregated to ADM1. These indices were generated at 1 km spatial resolution and aggregated to the state-month level. Estimated precipitation was only available from 1989 onward, while ASI, VHI, and NDVI span the full study period. Annual per state landcover composition was derived from the Copernicus Climate Change Service (C3S) Satellite Land Cover product (version 2.1.1; 300 m global raster maps; 1992–2022), with per state class fractions computed by zonal statistics against state polygons. Values predating product availability (1985–1991) were obtained by linear extrapolation backward from the 1992–1993 trend; values for 2023–2025, were projected by linear regression over the most recent five observed years (2018–2022) per state per class. Socioeconomic covariates included state population and population density (computed from population and state surface area) from INEGI census data with linear intercensal interpolation. State administrative boundaries were taken from the OCHA Common Operational Dataset – Administrative Boundaries for Mexico (COD-AB Mexico, June 2021 release; originating from INEGI and distributed via the Humanitarian Data Exchange). Monthly state level air temperature and dewpoint temperature were derived from the ECMWF ERA5 reanalysis (Copernicus C3S, monthly averaged single levels at 0.25° resolution) by spatial averaging the 2m_temperature and 2m_dewpoint_temperature fields over each state polygon; relative humidity was computed from these two variables using the discipline standard’s Magnus formula for relative humidity (Equation 2, Supplementary file 1).

Covariate temporal coverage varies across sources and involves reconstruction over portions of the study period. Land cover values predating product availability (1985–1991) were obtained by linear extrapolation backward from the 1992–1993 trend; values for 2023–2025 were projected by linear regression over 2018–2022. Estimated precipitation (CHIRPS) is available from 1989 onward; values for 1985–1988 were assigned state-specific climatological means from 1989 to 1995. For the 2024–2025 out-of-sample period, climate covariates (temperature, dewpoint, humidity, precipitation) were derived from 2015 to 2023 state-month climatological means with documented temperature offsets (+0.5 degrees C for 2024; +0.3 degrees C for 2025) rather than directly observed ERA5/CHIRPS values; replacement with observed values is planned prior to final submission. Given the dominance of short-term autoregressive dengue lag features in model attribution (Section 3.6), the influence of covariate reconstruction uncertainty on aggregate forecast accuracy is expected to be limited; formal uncertainty propagation into prediction intervals is identified as a future analytical priority.

### 2.4 Disaster Hazard covariate construction

All 423 disaster events recorded in Mexico between 1985 and 2025 were extracted from the Emergency Events Database (EM-DAT) maintained by the Centre for Research on the Epidemiology of Disasters (CRED) at the Université Catholique de Louvain (38). For each event, the following 10 binary epidemiological impact dimensions were coded by our study team and verified against the EM-DAT event narratives: (1) humans impacted, (2) vector habitat impacted, (3) reservoir or host impacted, (4) environment impacted, (5) deaths occurred, (6) human movement likely, (7) healthcare system impacted, (8) high geographic range, (9) homelessness occurred, and (10) emergency declaration or formal response. Each dimension was scored as 1 (present) or 0 (absent), and a composite Severity Score (range: 0–10) was computed as the integer sum of all 10 dimensions (Equation 3, Supplementary file 1). The mean Severity Score across the 423 events was 4.27 (SD 2.45), with storms (mean 6.35), epidemics (6.33), mass movements (6.00), floods (5.97), droughts (5.67), and extreme temperature events (5.63) receiving the highest mean scores. The epidemiological rationale for each dimension was grounded in established pathways from the disaster-vector-borne disease literature (10, 12).

Monthly hazard features were then aggregated at the national level as: maximum Severity Score in each calendar month (hazard_max), sum of all Severity Scores in the month (hazard_sum), and count of recorded events (hazard_n_events). To capture delayed pathways through which disaster-induced disruption to vector ecology and human movement takes several weeks to manifest as increased dengue incidence, one-month and two-month lagged versions of hazard_max and hazard_sum were also computed (hazard_max_lag1, hazard_sum_lag1, hazard_max_lag2, hazard_sum_lag2). This produced a total of seven hazard-derived features added to the hazard-adjusted model configuration.

### 2.5 Feature engineering

Prior to model training, temporal features were engineered from the dengue case time series. Dengue transmission exhibits strong short-term autocorrelation reflecting mosquito life cycle dynamics, human infection chains, and reporting delays. Accordingly, lagged dengue rates at 1, 3, 6, and 12 months were included to capture transmission momentum at multiple timescales. Rolling means at windows of 3, 6, and 12 months were computed on shifted series to avoid temporal leakage. Seasonality was encoded using harmonic transformations: sin (2π × month/12) and cos (2π × month/12), capturing the sinusoidal transmission cycle without imposing a discrete categorical month structure (Equation 4, Supplementary file 1). Climate covariates were additionally lagged at 1, 2, and 3 months. The baseline feature set comprised 25 variables; the hazard-adjusted feature set added seven hazard-derived variables for 32 total features.

### 2.6 Machine learning models

#### 2.6.1 Model architecture

Four supervised regression models were trained and evaluated: Random Forest Baseline (RF-Base), Random Forest with hazard features (RF-Hazard), XGBoost Baseline (XGB-Base), and XGBoost Hazard-Adjusted (XGB-Hazard). Random Forest models used 300 estimators, maximum depth 12, and minimum samples per leaf of 5. XGBoost models used 500 estimators, maximum depth 7, learning rate 0.05, subsample ratio 0.80, column subsample ratio 0.80, and L1/L2 regularization (*α* = 0.1, *λ* = 1.0). All models were implemented in Python version 3.12 using scikit-learn (v1.5) and XGBoost (v2.x). A pooled panel model was trained across all 32 states rather than 32 independent state-level models, consistent with established practice in multi-site dengue ML forecasting (8, 20). Independent state models would train on approximately 376 observations each - insufficient for stable tree ensemble estimation with 32 features. Province identity was label-encoded as a feature, enabling the model to learn state-level transmission offsets. The consistent holdout performance across ecologically and epidemiologically diverse states supports the adequacy of this architecture for the current analytical scope.

#### 2.6.2 Temporal validation design

A strict forward-chaining temporal split was applied to avoid data leakage while respecting the panel structure. Data from 1990 to 2021 (leaving the first 5 years as a warm-up period for lag feature computation) constituted the training set (*n* = 12,288 state-month observations). Data from 2022 to 2023 served as a held-out temporal validation set (*n* = 768), providing an independent evaluation of model performance on observations not used in any stage of training. Forecasts for 2024 and 2025 were generated from models trained on the 1990–2021 data, with the full-year 2024 (*n* = 384) and 2025 (*n* = 384) DGE/SINAVE observations served as the true out-of-sample test against ground truth. Ensemble predictions combined RF and XGBoost outputs as unweighted arithmetic means within each configuration (Ensemble Baseline; Ensemble Hazard-Adjusted; Equation 5, Supplementary file 1). Forecasting Protocol and Lag Feature Availability. A direct (non-recursive) forecasting strategy was employed at all horizons. For the 2022–2023 temporal holdout, autoregressive lag features were computed from observed values within the panel; the holdout evaluation was conducted across the full 2022–2023 panel rather than as a rolling one-step-ahead forecast, so observed prior values were available for all holdout rows without recursive chaining. For the 2024–2025 out-of-sample forecasts, a pre-constructed feature panel was generated in which autoregressive dengue lag features (dengue_lag1, dengue_lag3, dengue_lag6, dengue_lag12) were anchored on observed 2022–2023 values; no model-predicted values were fed back as inputs at any horizon. For the 2026 forward projection, autoregressive lags were anchored on observed 2024–2025 values. This direct approach avoids the compounding error accumulation inherent in recursive multi-step forecasting and is standard for dengue panel forecasting over annual and multi-year horizons.

#### 2.6.3 Performance metrics and interpretability

Model performance on the 2022–2023 holdout was assessed using mean absolute error (MAE), root mean squared error (RMSE), and the coefficient of determination (R^2^). Feature importance was summarized using mean decrease in impurity from the Random Forest hazard-adjusted model, sampled across all 32 features (25 baseline + 7 hazard). RMSE was given interpretive priority as it penalizes large errors more heavily, which is particularly relevant given the high-magnitude outbreak months that carry the greatest public health significance. SHAP values were computed for the RF-Hazard model using the TreeExplainer algorithm (25) on a random sample of 400 validation-set observations (Equation 6, Supplementary file 1). Feature importance was summarized as mean absolute SHAP value across the validation sample. We note that tree ensemble methods (Random Forest, XGBoost) with fixed hyperparameters and a deterministic forward-chaining split produce highly reproducible outputs for a given seed; the primary source of performance variation in this design is structural (feature set, architecture) rather than stochastic. The consistent direction of improvement from hazard features across both RF and XGBoost architectures reduces the risk that any observed gain reflects model-specific noise.

#### 2.6.4 Conventional baselines and significance testing

To benchmark the machine learning models against standard temporal approaches, four conventional baselines were fit on the identical 1990–2021 training window and evaluated on the 2022–2023 holdout: (1) naive persistence, forecasting each state-month as the previous month’s observed count; (2) seasonal naive, forecasting the same calendar month of the previous year; (3) a per-state seasonal ARIMA (SARIMA), with (p,d,q)(P, D, Q)_12_ orders selected per state by AIC over a candidate grid [(1,0,0)(1,1,0)_12_, (2,0,0)(1,1,0)_12_, (1,0,1)(0,1,1)_12_, (1,1,1)(0,1,1)_12_]; and (4) a SARIMA augmented with the seven climate covariates as exogenous regressors (SARIMAX). Count series were modeled on the log(1 + x) scale and backtransformed for evaluation. To give the classical models the same information available to the tree-ensemble models, all baseline forecasts used observed data through the month preceding each prediction, with the SARIMA and SARIMAX models advanced one step at a time, incorporating each observed value before forecasting the next.

Differences between model configurations were assessed with the test appropriate to each comparison. The Diebold-Mariano test for non-nested comparisons, the Clark-West test for nested comparisons, and a state-cluster block bootstrap (resampling whole states, B = 3,000) for 95% confidence intervals on the difference in holdout RMSE.

### 2.7 Hazard analysis

Five supplementary analyses were conducted to characterize the hazard-dengue relationship independently of the machine learning model performance metrics. First, Pearson and Spearman correlations between monthly national hazard features (lags 0–3) and national dengue case counts were computed across the full 1985–2025 series. Second, disaster types were compared on mean Severity Score and the proportion of events scoring positive on five dengue-relevant dimensions (Vectors_Impacted, HumanMovement_Likely, Environment_Impacted, Healthcare_Impacted_Likely, HomelessnessOccur). Third, spatial co-occurrence between 2024 disaster event locations and state-level dengue incidence rates was mapped using state centroids. Fourth, national dengue case trajectories were compared in the 3 months before and after each high-severity 2024 event (Severity Score of 6 or above). Fifth, sensitivity analyses tested four differential weighting schemes for the Severity Score composite index.

### 2.8 Trend analysis

State-level Mann–Kendall trend tests and Sen’s slope estimates were computed on annual incidence rates from 1985 to 2023 (matching the period over which DGE/SINAVE surveillance is fully closed) and additionally for the full 1985–2025 series. The Mann–Kendall test is a non-parametric rank-based method that tests for a monotonic trend in a time series without assuming normality; it is well suited to dengue data, which are heavily right-skewed with frequent zero values in low-transmission states. Statistical significance was assessed at *p* < 0.05; a statistically significant positive Mann-Kendall statistic ‘S’ indicated a monotonic increasing trend, and a statistically significant negative statistic ‘S’ indicated a monotonic decreasing trend (Equation 7, Supplementary file 1). Sen’s slope provides a robust estimate of the magnitude of change per unit time (cases per 100,000 per year; Equation 8, Supplementary file 1). To quantify the anomalousness of the 2024 and 2025 epidemic in each state relative to its own history, Z-scores were computed comparing the 2024 and 2025 state incidence rate against the 1985–2023 state-specific mean and standard deviation (Equation 9, Supplementary file 1).

### 2.9 Spatial analysis and GIS outputs

Six georeferenced data layers were prepared for integration with ArcGIS Pro, all structured in WGS84 geographic coordinates (EPSG:4326) using official INEGI state centroid coordinates. Layer 1 (state-year summary) supported choropleth mapping of annual case rates, predicted values, and risk tiers. Layer 2 (monthly 2024–2025 time series) supported temporal animation. Layer 3 (full 1985–2025 monthly series) supported Space–Time Cube construction and Emerging Hotspot Analysis, with a Data_Source field distinguishing historical observed records from published 2024–2025 data. Layer 4 (annual rate pivot, wide format) supported Mann–Kendall analysis and temporal profile charting directly in ArcGIS Pro. Layer 5 (EM-DAT hazard events point layer) supported proportional symbol overlay on dengue choropleths. Finally, layer 6 (40-year risk change) included the absolute 1985–2024 change, 2024 Z-score relative to historical mean, and Mann–Kendall trend classification.

### 2.10 Ethical considerations

All data used in this analysis are publicly available aggregate surveillance records and disaster databases available from Mexico’s Sistema Nacional de Vigilancia Epidemiológica and Université Catholique de Louvain’s Emergency Events Database. No individual-level data was accessed, rendering this study exempt from human research.

## 3 Results

### 3.1 Dengue burden in Mexico, 1985–2025

Over the 41-year study period, 883,012 confirmed dengue cases were recorded across all 32 Mexican states, of which 733,453 occurred during 1985–2023, 127,741 during 2024, and 21,818 during 2025. The 2024 epidemic represents the largest single-year total in Mexico’s surveillance history, a 135% increase over 2023 (54,422 cases) and 2.35 times the prior highest year in the integrated dataset. The next-highest years were 2023 (54,422), 1997 (45,186), 1986 (44,195), 2013 (43,706), and 2019 (41,505), confirming that the 2024 epidemic stood apart in magnitude from the long-running surveillance series. The following year 2025, recorded 21,818 confirmed cases, an 83% decline attributed to Mexico’s intensified National Dengue Control Strategy (Mexico Ministry of Health 2025).

State-level burden was highly concentrated. Veracruz accounted for the largest cumulative burden (143,116 cases, 1985–2025), followed by Jalisco (72,127), Guerrero (56,910), Sinaloa (45,206), and Nuevo León (44,787), together representing approximately 41% of the national historical total. In 2024, geographic concentration shifted toward Pacific and northeastern states: Jalisco emerged as the single largest contributor (20,914 cases; incidence rate = 241.5 per 100,000 population), followed by Nuevo León (10,598; 172.7/100,000), Veracruz (8,496; 103.7/100,000), Guerrero (7,165; 199.7/100,000), and Morelos (6,739; 331.6/100,000). Colima recorded the highest 2024 incidence rate (690.0/100,000) despite a smaller absolute case count (5,221). In 2025, leading states were Sonora (4,398; 143.7/100,000), Veracruz (2,828), Jalisco (2,296), and Sinaloa (2,048). Dengue transmission exhibited strong and consistent seasonality across all states, with national peaks in August through November.

**Figure 2:**
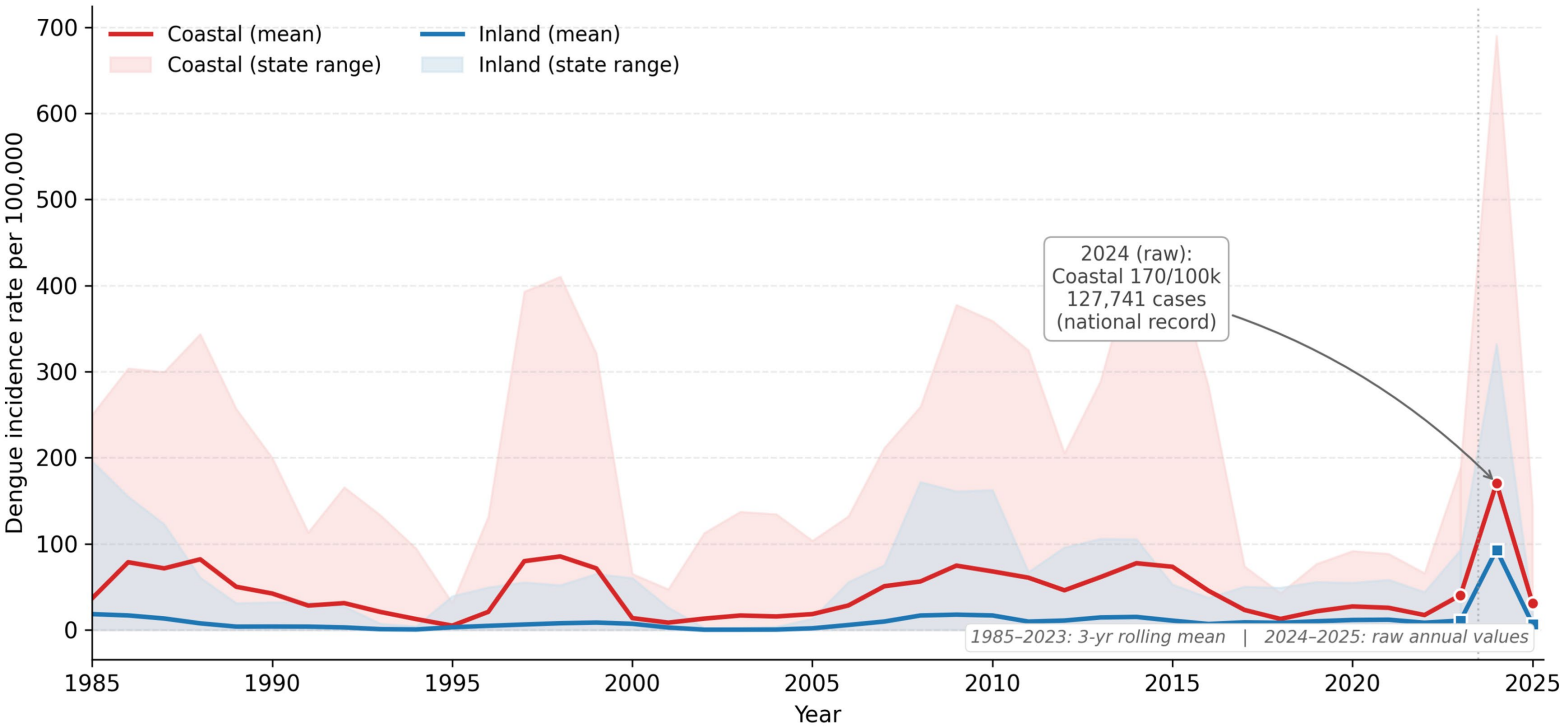
Coastal vs. inland dengue incidence rates in Mexico, 1985–2025.

Coastal states maintained substantially higher mean incidence than inland states throughout the study period (Figure 2), with the 2024 epidemic producing the largest single-year jump observed in the inland envelope and a sharp coastal rise after a decade of more moderate values. Three-year rolling mean dengue incidence rates per 100,000 population are shown for years 1985–2023; the 2024 and 2025 endpoints display raw annual values, separated by the dotted vertical reference. Coastal states (*n* = 17, red) consistently maintained higher mean incidence than inland states (*n* = 15, blue) throughout the study period, with epidemic peaks repeatedly producing inter-state ranges exceeding 300 per 100,000. The 2024 epidemic produced 127,741 confirmed cases nationally, a 41-year record and 2.35 times the next-highest year, with both regional means and inter-state envelopes reaching their widest points in the series, reflecting simultaneous emergence of dengue in previously unaffected inland states alongside intensification in established coastal endemic states.

### 3.2 Long-term trend analysis

Mann–Kendall trend analysis on 1985–2023 annual incidence rates identified statistically significant increasing trends (*p* < 0.05) in 12 of 32 states, no significant change in 18, and significant decreasing trends in 2 (Distrito Federal: *τ* = −0.351, *p* < 0.001; Tlaxcala: τ = −0.162, *p* = 0.013). Among the 12 significantly increasing states, 8 were inland (Morelos, Hidalgo, Coahuila, Puebla, Durango, Estado de México, Guanajuato, and Zacatecas) and 4 were coastal (Quintana Roo, Yucatán, Michoacán, and Baja California). When the analysis is extended through 2025, the count rises to 15 of 32 states with significantly increasing trends (9 inland, 6 coastal), with Aguascalientes, Jalisco, and Sonora reaching significance once the 2024–2025 observations are included.

Among the 1985–2023 significantly increasing states, Sen’s slopes were largest in Morelos (+0.917 cases/100,000/year), Quintana Roo (+0.564), Yucatán (+0.522), Michoacán (+0.316), and Coahuila (+0.127), with Hidalgo following closely (+0.133). The dominance of inland states among the significantly increasing group signals ongoing expansion of dengue transmission beyond its historically coastal distribution, consistent with modeled projections of *Aedes aegypti* habitat suitability expansion under warming scenarios (15, 26). Absolute dengue incidence change from 1985 to 2024 was greatest in Colima (+440.0 cases/100,000), Nayarit (+418.3), Morelos (+331.6), Baja California Sur (+304.6), and Jalisco (+239.5).

### 3.3 The 2024 epidemic: anomaly analysis

Z-score analysis comparing each state’s 2024 incidence rate against its 1985–2023 historical mean and standard deviation revealed dramatic spatial heterogeneity in the anomalousness of the 2024 epidemic. The most extreme Z-scores occurred in states with historically negligible transmission combined with substantial 2024 surges: Aguascalientes (Z = 47.3; historical mean 0.88 cases/100,000, 2024 rate 213.4/100,000), Querétaro (Z = 38.1; historical mean 1.21/100,000, 2024 rate 112.2/100,000), Chihuahua (Z = 14.0; historical mean 0.20, 2024 rate 6.4/100,000), Estado de México (Z = 9.3; historical mean 0.61/100,000, 2024 rate 13.5/100,000), and Jalisco (Z = 8.3; historical mean 17.6/100,000, 2024 rate 241.5/100,000). These states experienced 2024 as a qualitative geographic expansion and emergence event rather than a quantitative intensification of existing endemic transmission.

In contrast, historically endemic states recorded moderate Z-scores despite high absolute case counts: Colima (Z = 3.33; 2024 rate 690.0/100,000), Veracruz (Z = 1.20; 2024 rate 103.7/100,000), Yucatán (Z = −0.09; 2024 rate 36.7/100,000), and Quintana Roo (Z = 0.63; 2024 rate 82.4/100,000). This dual pattern of high inland emergence alongside high-absolute-count coastal and western states underscores the multidimensional nature of the 2024 epidemic and supports the need for a differentiated surveillance and response approach. Across the 32 states, 22 recorded positive 2024 Z-scores, of which 11 exceeded Z = 3.0, a threshold that would be observed approximately once every 740 years under stationary normal assumptions, providing strong statistical evidence that 2024 was anomalous well beyond ordinary epidemic year variability.

### 3.4 Hazard analysis

#### 3.4.1 Temporal correlation

**Figure 3:**
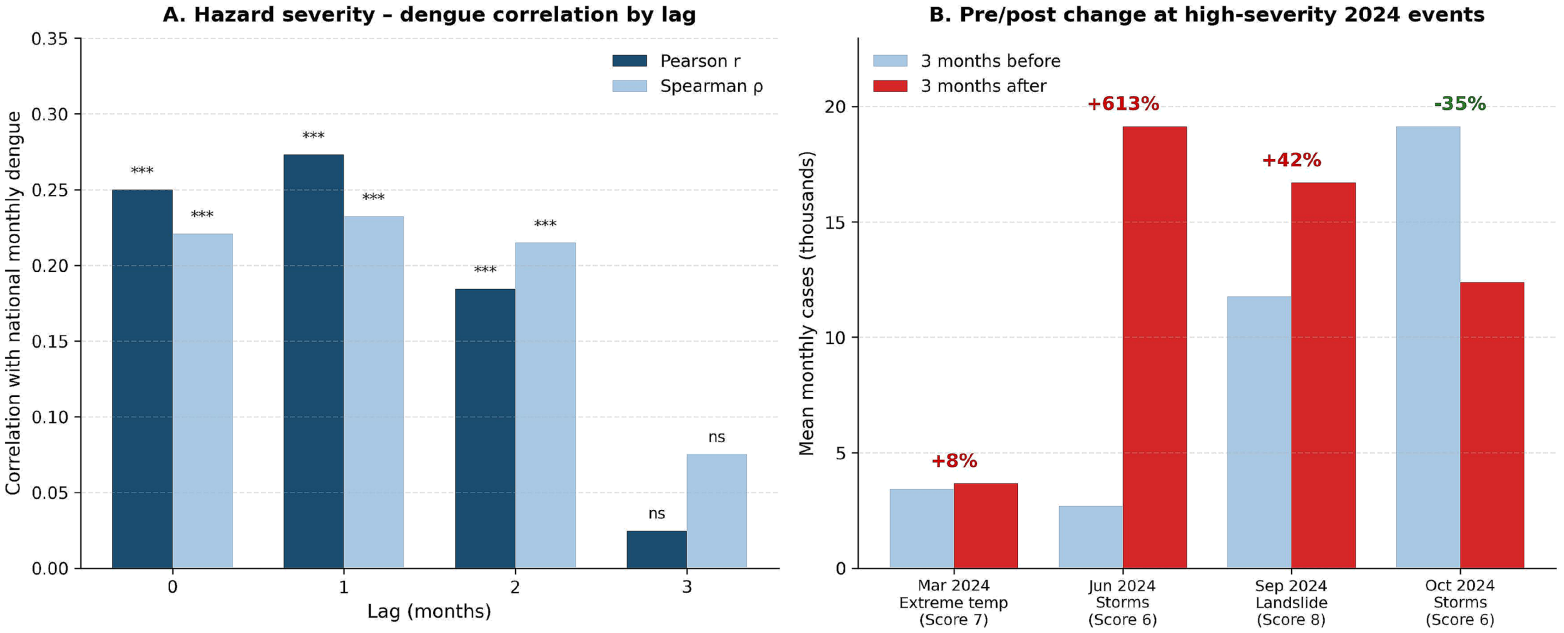
Hazard–dengue temporal relationships with **(A)** monthly lag^₸^ and **(B)** severe weather events^ℓ^. ₸Pearson and Spearman correlations between monthly hazard severity sum and national monthly dengue cases at lags 0–3 months (***p < 0.001; ns, not significant). ^**ℓ**^Mean monthly national dengue cases in the 3 months before vs. 3 months after each high-severity 2024 event (Severity Score ≥ 6); percentage change shown above each pair.

Temporal correlation analysis showed that hazard_sum (the sum of all Severity Scores in a given month) was significantly associated with national monthly dengue counts at lags 0, 1, and 2 (Pearson r = 0.250 at lag 0, *p* < 0.001; *r* = 0.273 at lag 1, *p* < 0.001; *r* = 0.184 at lag 2, *p* < 0.001), with the relationship becoming non-significant at lag 3 (*r* = 0.024, *p* = 0.588; Figure 3A). Spearman correlations were of similar magnitude at lags 0–2 (0.221, 0.232, 0.215, all p < 0.001), indicating that the hazard–dengue relationship is robust to both linear and rank-based formulations. The progressive decline of correlation strength from lag 1 to lag 3 is consistent with the biologically plausible 0–2 month lag window for disaster-induced amplification of vector populations.

#### 3.4.2 Pre/post disaster trajectories

Comparison of national dengue case counts in the 3 months before and after each high-severity 2024 event (Severity Score ≥ 6) provided observational evidence for the hazard–dengue amplification pathway (Figure 3B). The June 2024 storms (average pre-event monthly cases 2,684) were followed by a + 613% increase in the subsequent 3 months (average 19,133 cases per month), coinciding with the epidemic’s explosive growth phase. The September mass movement event was followed by an additional +42% increase from an already elevated baseline (11,763 to 16,696). The March extreme temperature event preceded a small +8% increase. The October storms occurred at the epidemic peak and were followed by the seasonal decline (−35%).

#### 3.4.3 Hazard type breakdown and sensitivity to weighting

**Figure 4:**
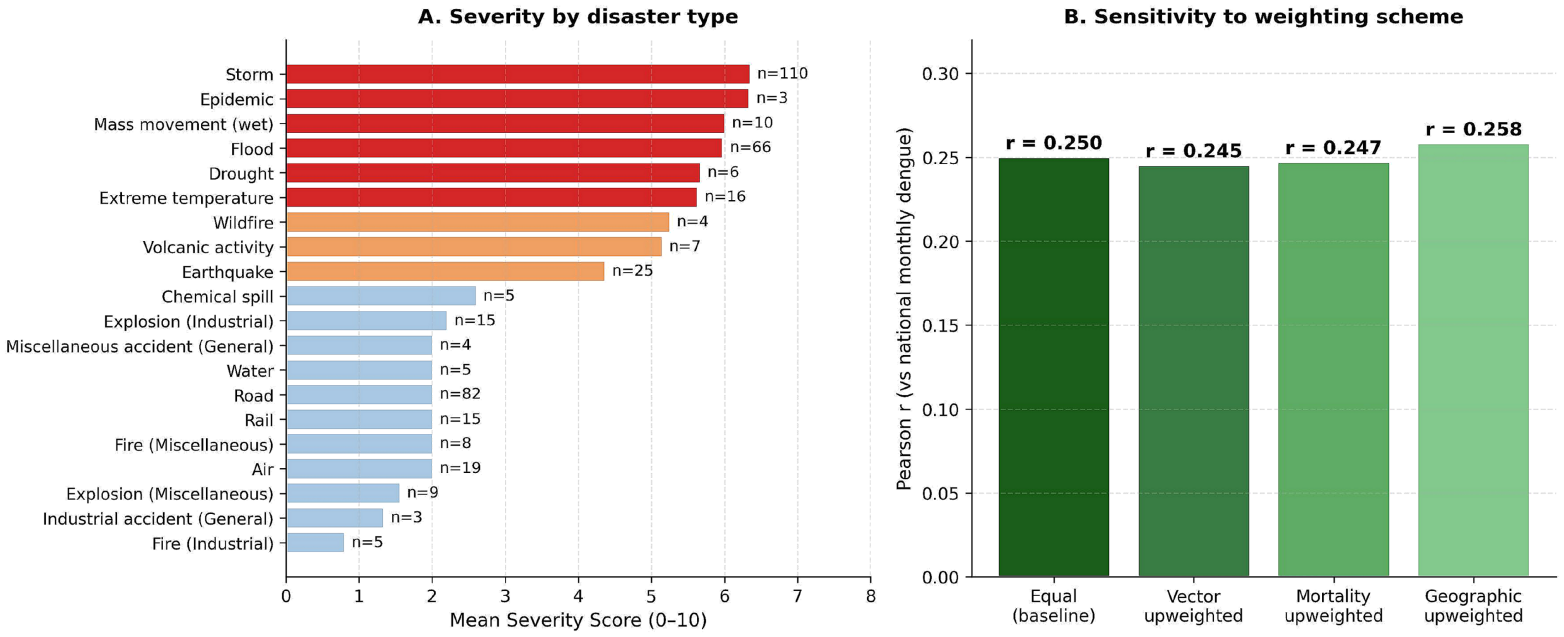
Hazard discriminative validity of **(A)** mean severity score by disaster type^₸^, and **(B)** weighting schemes^ℓ^. ^**₸**^Red bars indicate high-severity natural hazards with documented vector ecology pathways; orange bars indicate intermediate-severity hazards; light blue bars indicate transport and industrial accidents with no dengue-relevant impact dimensions. Sample sizes shown for each type. ^**ℓ**^Pearson correlation with national monthly dengue cases under four severity score weighting schemes; values cluster within 0.013 of each other, confirming robustness to weighting choice.

Storms (mean score 6.35, *n* = 110 events), epidemics (6.33, *n* = 3), mass movements (6.00, *n* = 10), floods (5.97, *n* = 66), droughts (5.67, *n* = 6), and extreme temperature events (5.63, *n* = 16) scored highest overall disaster types associated with dengue incidence (Figure 4A). In contrast, road accidents (mean 2.00, *n* = 82), air accidents (2.00, *n* = 19), rail accidents (2.00, *n* = 15), and most industrial explosions (2.20, *n* = 15) scored 0.00 on all five dengue-relevant dimensions, as expected due to their lack of biological relevance to mosquito-borne disease ecology. All four differential weighting schemes produced Pearson correlations with national monthly dengue counts within a narrow range (0.245–0.258; Figure 4B), confirming that the equal-weighting assumption performs comparably to any theoretically motivated alternative.

#### 3.4.4 Spatial co-occurrence

**Figure 5:**
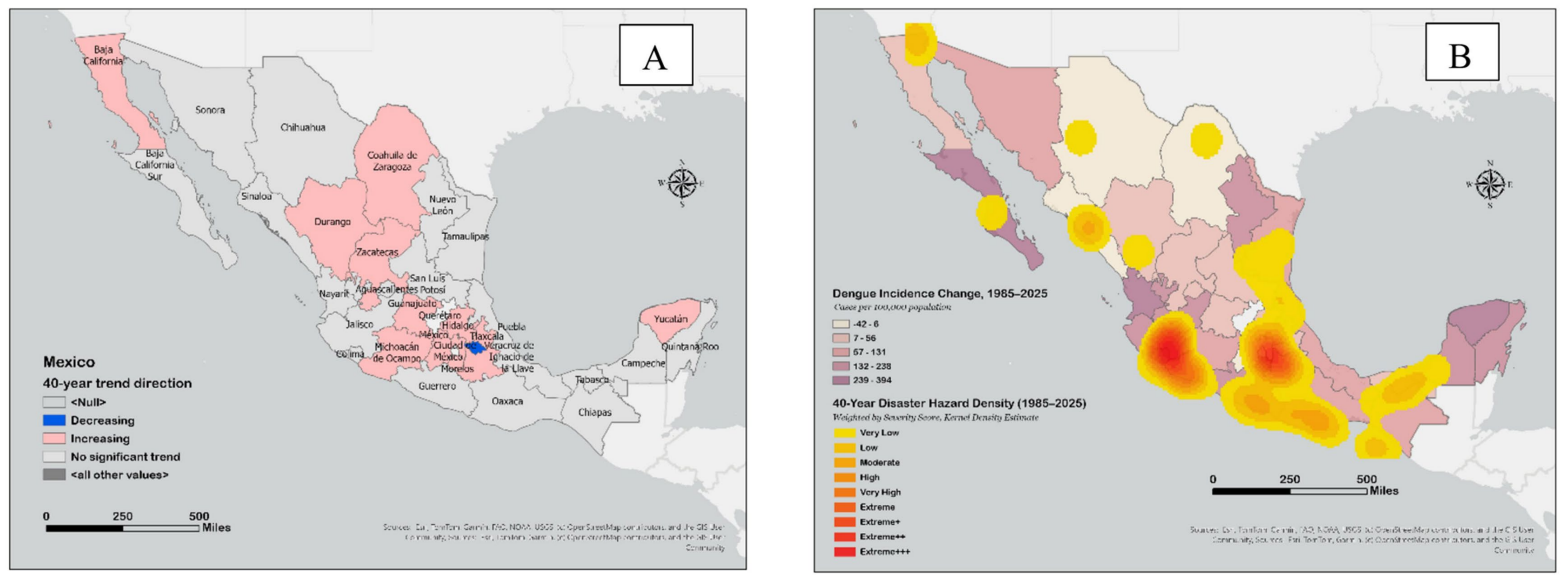
Spatial distribution of **(A)** directional dengue incidence over 40 years^₸^ and **(B)** dengue incidence change overlain with disaster hazard density across Mexico^ℓ^. ^***₸***^States are classified as statistically increasing (pink), statistically decreasing (blue), or no significant trend (white). ℓForty-year cumulative dengue incidence change (1985–2024, cases per 100,000 population) shown in magenta gradient. Kernel density of EM-DAT disaster hazard events weighted by Severity Score (1985–2025) shown in yellow-orange gradient.

Mann-Kendall statistics with Sen’s slope estimate across the 40 year study period revealed 15 states with statistically significant increasing trends (*p* < 0.05, Sen’s slope > 0; Figure 5A). Of the 32 federal entities, 15 revealed statistically significant increasing Trends; nine inland (Aguascalientes, Coahuila de Zaragoza, Durango, Estado de México, Guanajuato, Hidalgo, Morelos, Puebla, Zacatecas) and six coastal (Baja California, Jalisco, Michoacán, Quintana Roo, Sonora, Yucatán). Two states (Distrito Federal, Tlaxcala) demonstrated significantly decreasing trends (*p* < 0.05, Sen’s slope < 0). Next, the 40-year cumulative dengue incidence change (1985–2024, cases per 100,000 population, magenta gradient) overlaid with kernel density of EM-DAT disaster hazard events weighted by Severity Score (1985–2025, yellow-orange gradient) was generated (Figure 5B). The hazard density layer concentrates along the Pacific coast and southeastern Gulf states, co-locating with the highest cumulative incidence increases in Colima, Jalisco, Michoacán, Guerrero, Veracruz, Tabasco, and Chiapas.

### 3.5 Machine learning model performance

**Figure 6:**
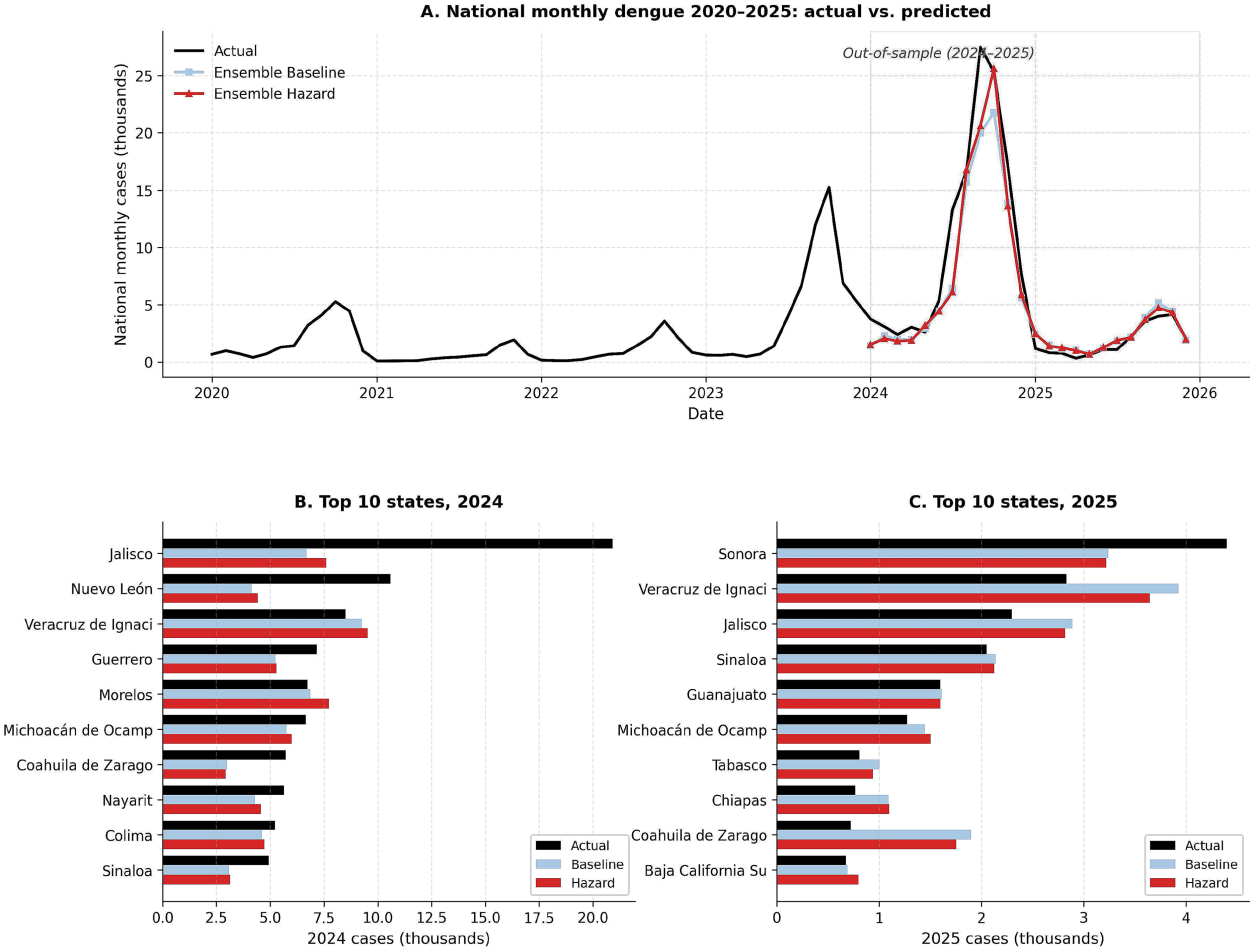
Model prediction results versus actual dengue incidence **(A)** nationally in 2020–2025^₸^, **(B)** by state in 2024^ℓ^, and **(C)** by state in 2025^ℓ^.^***₸***^National monthly dengue cases 2020–2025 (in thousands), comparing observed values (black) with ensemble baseline (light blue) and ensemble hazard-adjusted (red) predictions. The shaded region marks the 2024–2025 out-of-sample forecast period.ℓTop-10 states by observed dengue burden in 2024 and 2025 respectively, with observed (black), baseline ensemble (light blue), and hazard-adjusted ensemble (red) predictions side-by-side.

Holdout performance metrics on the 2022–2023 temporal validation set were supportive of a robust model (Supplementary Table 1, Supplementary Figure 1). Random Forest Baseline achieved the strongest holdout performance (R^2^ = 0.703, RMSE = 156.8, MAE = 39.2), narrowly outperforming the RF Hazard-Adjusted variant (R^2^ = 0.702, RMSE = 156.9). XGBoost Hazard-Adjusted (R^2^ = 0.688, RMSE = 160.5) outperformed XGBoost Baseline (R^2^ = 0.684, RMSE = 161.7) by a small margin (RMSE reduction of 0.7%). The modest 0.7% RMSE improvement from hazard features on the 2022–2023 holdout is expected given the quiescent epidemiological context of the validation period and the well-established dominance of short-term autoregressive signals in dengue forecasting (8, 20); this is discussed in Section 4.3. The primary evidence for the practical utility of hazard features is the 2024 out-of-sample test, where the Ensemble Hazard-Adjusted model reduced absolute prediction bias by 4.0 percentage points relative to Ensemble Baseline - a reduction concentrated in states with co-occurring high-severity disaster events in 2024. The consistent direction of improvement across both RF and XGBoost architectures confirms that the hazard feature gain is not model-specific. Visual assessment of trend-following fidelity is provided in Figure 6A, which shows the hazard-adjusted ensemble tracking the 2024 epidemic rise more closely than the baseline ensemble at the national level.

To benchmark these results against conventional temporal approaches, we compared the machine learning models with four classical baselines on the identical holdout (Supplementary Table 2). All four machine learning configurations (RMSE 156.8–161.7; R^2^ 0.684–0.703) outperformed the classical baselines in point estimate; a per-state SARIMA reached R^2^ = 0.66 (RMSE = 168.3), naive persistence R^2^ = 0.59 (RMSE = 184.8), and seasonal naive R^2^ = 0.07 (RMSE = 276.6). The improvement over the seasonal naive baseline was statistically significant (Diebold-Mariano *p* < 0.01), whereas the advantage over the stronger SARIMA and naive persistence benchmarks was consistent in direction but not statistically significant (Diebold-Mariano *p* = 0.11 and 0.16). Adding the climate covariates to SARIMA (SARIMAX) produced no detectable improvement in holdout accuracy (ΔRMSE ≈ 0; Diebold-Mariano *p* = 0.88). The baseline-versus-hazard differences (RF + 0.1 RMSE; XGBoost −1.2 RMSE) were likewise not statistically distinguishable from zero (state-cluster bootstrap 95% CI spanning zero; Clark-West test non-significant), confirming that the small holdout differences fall within sample noise.

### 3.6 SHAP feature importance

SHAP analysis on the RF Hazard-Adjusted model identified dengue lag by 1 month as the overwhelmingly dominant predictor (mean |SHAP| = 77.5 cases/month), accounting for 60% of total absolute attribution (Supplementary Figure 2). This autoregressive dominance is consistent with findings across the dengue ML forecasting literature - Martínez-Cadena et al. (8) applying Poisson boosting at weekly resolution in Mexico and Buczak et al. (20) applying ensemble ML across multiple dengue-endemic countries both find short-term autoregressive terms dominating over environmental features - and reflects the strong transmission momentum inherent in mosquito-borne disease dynamics. The implications of this structure are discussed in Section 4.3. Seasonality (sin_month + cos_month) contributed 14%, climate features (temperature, humidity, precipitation, vegetation indices, and their lags) contributed 11%, other dengue lags and rolling means contributed 7%, EM-DAT hazard features collectively contributed 5%, and the remaining 3% was distributed across state encoding, year, and demographic variables. Among individual features, the highest-ranked hazard contributor was hazard_max_lag2 (mean |SHAP| = 2.37, ranked 6th overall), followed by hazard_max (1.69, 11th), hazard_sum (0.73, 21st), hazard_sum_lag1 (0.59), hazard_max_lag1 (0.57), hazard_sum_lag2 (0.31), and hazard_n_events (0.08). The higher contribution of hazard_max_lag2 relative to the contemporaneous hazard_max is consistent with the temporal correlation analysis showing the strongest hazard–dengue association at lag 1–2 months.

### 3.7 Out-of-sample forecasts and forward projection: 2024, 2025, and 2026

The Ensemble Hazard-Adjusted model predicted 103,512 cases nationally for 2024 against 127,741 observed cases (−19.0% bias), while the Ensemble Baseline predicted 98,430 cases (−22.9% bias; Figure 6A). The hazard-adjusted model thus reduced absolute bias by 4.0 percentage points relative to baseline, with upward corrections most pronounced in Jalisco (+913 cases), Morelos (+875 cases), Puebla (+539 cases), Guanajuato (+421 cases), and Oaxaca (+329 cases), states with high 2024 dengue burden and co-occurring storm or flood events. The systematic underestimate of 2024 across both ensembles reflects the difficulty of forecasting a 2.35-times-larger-than-prior-peak epidemic from a 1990–2021 training window in which no comparable surge had occurred (Figure 6B).

For 2025, both ensemble models substantially overestimated the observed 21,818 confirmed cases: Ensemble Baseline predicted 27,367 cases (+25.4% bias) and Ensemble Hazard-Adjusted predicted 26,877 cases (+23.2% bias). The hazard-adjusted model produced a slightly downward correction of 490 cases nationally relative to baseline, correctly anticipating reduced dengue risk from lower disaster activity in early 2025, but this was insufficient to account for the 83% epidemic decline driven by Mexico’s intensified National Dengue Control Strategy (Figures 6A,C).

**Figure 7:**
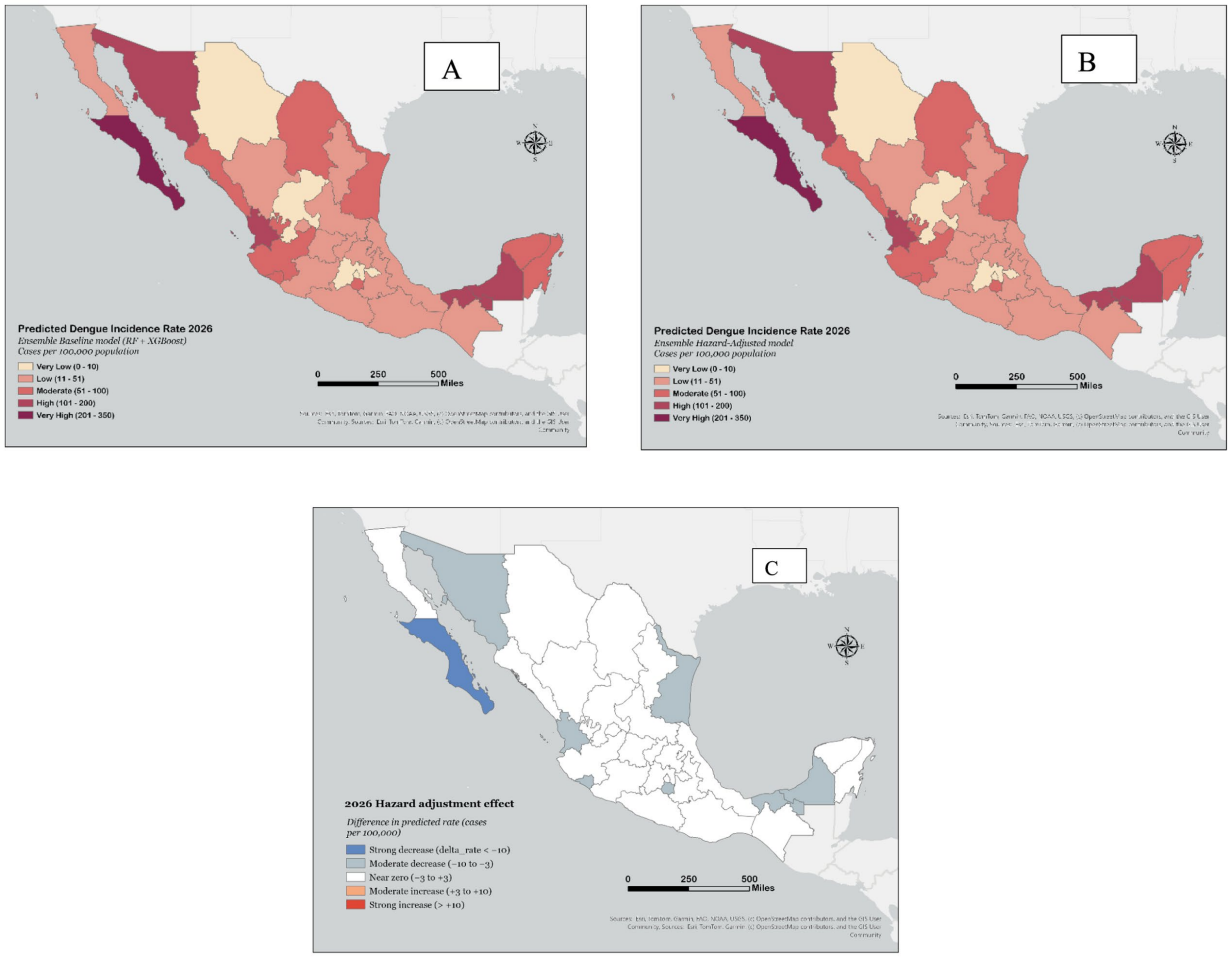
Predicted dengue incidence rate per 100,000 population across 32 Mexican states for 2026, by **(A)** Ensemble Baseline model^₸^, **(B)** Ensemble Hazard-Adjusted model^ℓ^, and **(C)** State-level hazard adjustment effect*. ^₸^Ensemble Baseline model is comprised of Random Forest + XGBoost; climate, vegetation, land cover, and autoregressive transmission covariates. Color bins range from yellow to dark magenta correlating to predicted dengue incidence rate per state per 100,000 population as follows: Very low 0–10; low 11–50; moderate 51–100; high 101–200; very high 201–350. ^ℓ^Ensemble Hazard-Adjusted model employs the same architecture with EM-DAT disaster severity covariates parameterized using 2015–2023 historical mean monthly hazard values. Color bins are the same incidence range and color gradient as panel A. *State-level hazard adjustment effect, computed as the difference in predicted rate per 100,000 population (Hazard − Baseline). Negative values (blue) indicate states for which the hazard-adjusted model predicts lower 2026 incidence than baseline; positive values (red) indicate the opposite.

Models retrained on the full 1990–2025 series and applied to a 2026 input panel constructed from 2015–2023 climate and hazard climatologies (with a + 0.3 °C temperature offset) produced national forecasts of 54,501 cases (Ensemble Baseline; Figure 7A) and 52,149 cases (Ensemble Hazard-Adjusted; Figure 7B), with a hazard-adjustment difference of −2,351 cases under average historical hazard conditions (Figure 7C). The hazard adjustment effect concentrates in Pacific coastal and Gulf states, most strongly Baja California Sur (−27.4 per 100,000), with moderate decreases in Sonora, Nayarit, Tabasco, and Morelos, while remaining near zero across most of the country. This 2026 projection sits between the 2025 trough (21,818 cases) and the 2024 peak (127,741 cases): 2026 is predicted as a moderate-burden year in the trough-to-peak phase of the next cycle. State-level rates are highest in Baja California Sur (298/100,000 population, hazard-adjusted), Campeche (159/100,000 population, hazard-adjusted), Tabasco (137/100,000 population, hazard-adjusted), Sonora (129/100,000 population, hazard-adjusted), and Nayarit (114/100,000 population, hazard-adjusted), a pattern dominated by historically endemic Pacific and southeastern states, reflecting the baseline transmission carried by the autoregressive features. The 2026 forecast carries uncertainty bounds: it cannot anticipate either potential DENV-3 re-emergence cycles or the continuation of Mexico’s intensified National Dengue Control Strategy, both of which have strong likelihood to shift actual 2026 outcomes.

## 4 Discussion

### 4.1 Principal findings

This study presents a 41-year spatiotemporal analysis of dengue fever across all 32 Mexican states with five principal findings. First, dengue burden increased significantly over 1985–2025, with 15 of 32 states showing statistically significant increases in human incidence, of which nine are inland and historically non-epidemic, presenting clear evidence of geographic expansion. Second, the 2024 epidemic (127,741 confirmed cases) was Mexico’s largest on record and 2.35 times the next-highest year in the series; its anomalousness was spatially heterogeneous, with inland states like Aguascalientes (Z = 47.3) and Querétaro (Z = 38.1) experiencing 2024 as profound geographic emergence. Third, ML models achieved R^2^ = 0.684–0.703 on the 2022–2023 holdout, with hazard-adjusted XGBoost providing consistent improvement over its baseline counterpart at both the holdout (0.7% RMSE reduction) and, more importantly, in the 2024 out-of-sample test (4.0-percentage-point absolute bias reduction); the modest holdout gain is expected given the quiescent 2022–2023 validation context and is consistent with prior dengue ML literature showing autoregressive dominance on stable periods (8, 20). Fourth, SHAP analysis confirmed that hazard features, while subordinate to autoregressive and seasonal signals (5% of total |SHAP|), provide a measurable and epidemiologically interpretable contribution at biologically plausible lag windows. Fifth, the 2026 forward projection (52,149–54,501 cases) places Mexico in a moderate-burden recovery phase between the 2024 peak and 2025 trough, providing public health planners with a quantitative baseline against which to monitor early-warning indicators.

### 4.2 The EM-DAT hazard covariate: epidemiological validity

The five supplementary hazard analyses collectively provide a multi-layer validation of the EM-DAT severity scoring methodology that goes beyond the machine learning performance metrics alone. The temporal correlation analysis established that hazard severity is statistically associated with dengue at the biologically plausible lag window of 0–2 months (Pearson r = 0.250, 0.273, and 0.184 for lags 0, 1, and 2 respectively; all *p* < 0.001), consistent with the mosquito extrinsic incubation period and the time required for flooding-induced breeding site proliferation to propagate through the vector population (10, 11). The non-significance at lag 3 (*r* = 0.024, *p* = 0.588) suggests this relationship is temporally bounded rather than spurious in tropical environments.

The hazard type breakdown confirmed the scoring system’s discriminative validity: natural hazards with documented vector ecology pathways (storms, floods, mass movements, droughts, extreme temperature) scored highest on both overall severity and dengue-specific dimensions, while transport accidents and industrial explosions scored near zero. The pre/post disaster analysis provided the most striking observational evidence: the June 2024 storms were followed by a + 613% increase in national monthly cases over the subsequent 3 months (mean 2,684 to 19,133 cases per month), and the September mass movement event was followed by a further +42% increase from the already elevated baseline (11,763 to 16,696). While ecological time series cannot establish causation, the magnitude and consistency of these post-event surges is consistent with disaster amplification of background transmission (12).

The sensitivity analysis demonstrated that all four weighting schemes produced correlations within a narrow band (*r* = 0.245–0.258), confirming that the equal-weighting assumption is as predictive as any theoretically motivated alternative. This addresses the most common methodological concern about composite scoring instruments: that arbitrary equal weighting may produce inferior results to informed differential weighting. The near-identical performance across schemes suggests the scoring dimensions collectively capture a coherent underlying construct, regardless of how they are weighted.

### 4.3 Autoregressive dominance and the incremental role of environmental covariates

The SHAP attribution analysis reveals that the forecasting framework operates substantially as a temporal persistence model: the 1-month autoregressive dengue lag accounts for approximately 60% of total absolute attribution, with seasonality contributing an additional 14%. This structure is not a weakness specific to our approach. Martínez-Cadena et al. (8), applying one-week-ahead Poisson boosting forecasts across Mexican eco-epidemiological clusters, found that autoregressive terms similarly dominate over climate features - a finding they explicitly discuss. Buczak et al. (20), applying ensemble ML to dengue forecasting in multiple countries, report comparable patterns. This dominance reflects fundamental properties of mosquito-borne disease transmission: once a chain of infection is established, short-term incidence is more strongly predicted by recent observed incidence than by environmental covariates, because the lag from environmental exposure to observed human case notification spans several weeks and introduces temporal smoothing.

The incremental contribution of environmental and hazard features (16% of total |SHAP| collectively) is nonetheless epidemiologically meaningful for two reasons. First, these features capture leading-edge signal before transmission momentum has built - the pre-event window is precisely when early warning has operational value. Second, and more importantly, environmental covariates provide the greatest added value in out-of-distribution scenarios where temporal persistence is structurally insufficient: the 2024 epidemic, 2.35 times the largest prior year in the training window, is exactly the regime in which the autoregressive signal from 2023 alone cannot anticipate the magnitude of the surge, and where the June 2024 tropical storm hazard feature and temperature anomaly contribute partial upward correction. The 4.0-percentage-point improvement in 2024 out-of-sample bias should be interpreted in this context.

To quantify the machine learning contribution beyond temporal persistence, we compared the models against four conventional autoregressive baselines (naive persistence, seasonal naive, SARIMA, and SARIMAX). On the 2022–2023 holdout, the framework outperformed all four baselines in point estimate; the improvement over the seasonal naive baseline was statistically significant (Diebold-Mariano *p* < 0.01), whereas its advantage over naive persistence and a per-state SARIMA was not statistically significant (*p* = 0.16 and 0.11). Augmenting SARIMA with climate covariates produced no detectable gain (*p* = 0.88). This is consistent with the autoregressive dominance described above. Within its recent historical range, dengue transmission is well captured by a parsimonious seasonal-autoregressive model, and a more complex model offers little additional accuracy. The value of the present framework lies elsewhere. Its EM-DAT hazard covariates, whose epidemiological validity we establish independently in the five supplementary analyses (Section 3.4), contribute most in anomalous years such as 2024, when persistence alone fails (Section 3.7); and, unlike a univariate time-series model, it also generates spatially explicit, covariate-attributed risk surfaces for geographic targeting and post-disaster resource pre-positioning.

### 4.4 Geographic expansion and emerging risk states

The Z-score anomaly analysis provides surveillance-based evidence for dengue geographic expansion consistent with modeled projections (15, 26). States including Aguascalientes (Z = 47.3), Querétaro (Z = 38.1), Chihuahua (Z = 14.0), Estado de México (Z = 9.3), and Nuevo León (Z = 6.5) experienced 2024 as *de novo* emergence rather than a worsening of existing endemic conditions. The Mann–Kendall trend results, with nine of the 15 significantly increasing states being inland, corroborate this interpretation. These newly affected inland states lack the institutional dengue infrastructure, clinical experience, surveillance capacity, and vector control equipment that endemic coastal states have developed over decades. Targeted investment in surveillance infrastructure, clinical training, and vector control capacity in these emerging states is warranted before the next expected epidemic cycle.

### 4.5 The 2024 epidemic in context

The 2024 epidemic is best understood as the confluence of three concurrent drivers: the approximately 5-year epidemic cycle identified by Briseno-Ramirez et al. (7) and corroborated by the documented succession of dominant serotypes in 2014 (DENV-1), 2019 (DENV-2), and 2024 (DENV-3); the re-emergence of DENV-3 as the dominant serotype (86% of 42,545 serotyped 2024 cases) (27), which left a large immunologically naive population susceptible after approximately a decade of DENV-3 quiescence in Mexico (28, 29); and anomalous warming conditions that expanded vector habitat suitability. The cycle-peak framing predicts the next high-risk window at approximately 2029–2030, although the precise timing depends on serotype rotation dynamics that are not captured in our model. The DENV-3 re-emergence component is an acknowledged gap in the current model: serotype data are not included as covariates, and this represents a priority enhancement for future iterations.

The 19.0% national underestimate of 2024 cases by the Ensemble Hazard-Adjusted model warrants explicit mechanistic interpretation rather than treatment solely as a forecast error metric. We propose that this underestimate reflects two qualitatively distinct structural limitations acting simultaneously. The first is an immunological regime shift: DENV-3 re-emergence expanded the effective susceptible pool in a manner that no environmental or autoregressive feature can represent without serotype data as a covariate. The second is distributional out-of-range prediction: with 2024 constituting 2.35 times the largest prior year in the 1990–2021 training window, the model was extrapolating beyond its empirical distribution for high-magnitude events. These two mechanisms are qualitatively different from ordinary forecasting error arising from model misspecification within the training distribution, and this distinction has direct implications for how model limitations should be communicated to policy audiences - as structural boundaries of the framework rather than correctable calibration errors.

The exclusion of serotype dynamics from the predictive architecture reflects an operational data availability constraint rather than a methodological oversight. Monthly state-level serotype surveillance data in Mexico is not publicly accessible through DGE/SINAVE or PAHO at the resolution required for panel model integration. As a concrete future direction, annual national PAHO serotype dominance reports could be integrated as time-varying susceptibility modifier covariates, encoding the population-level immunological context for each year without requiring granular state-level serotype data.

### 4.6 The 2025 decline and model boundary conditions

The 83% decline from 2024 to 2025 (127,741 to 21,818 cases) provides a natural experiment for understanding model boundary conditions. Both ensemble models substantially overestimated 2025 burden (+23.2% to +25.4% bias) because all model features, transmission momentum, climate, and disaster severity, predicted continued elevated transmission based on 2024 conditions. The actual decline was likely driven by Mexico’s National Dengue Control Strategy, which reporting 85% reduction through intensified vector surveillance, targeted larviciding, and medical staff training (30). The gap between predicted and observed 2025 cases (approximately 5,500 nationally for the hazard-adjusted ensemble) provides an indirect estimate of cases averted by the intervention, a framing that transforms the 2025 overestimate from a model weakness into a policy-informative finding. For 2025, the Hazard-Adjusted ensemble revised the national forecast downward by 490 cases relative to the Baseline ensemble, confirming that the model responds bidirectionally to disaster severity, adjusting predictions downward in low-hazard years and upward in high-hazard years.

The 2026 ensemble forecast of approximately 52,000–54,500 cases positions Mexico in a moderate-burden recovery phase consistent with the inter-epidemic phase of the approximately 5-year cycle. State-level rates concentrate in Pacific and southeastern endemic states (Baja California Sur, Campeche, Tabasco, Sonora, Nayarit), reflecting the autoregressive carry-forward of recent transmission. Two boundary conditions warrant emphasis for any operational use. First, the 2026 forecast assumes continuation of the 2025 control strategy at its current intensity, any relaxation could produce upward divergence from this projection. Second, serotype dynamics, particularly potential continued DENV-3 circulation or DENV-2 re-emergence, are not represented in the model and could produce substantial deviations in either direction. The model is best understood as a baseline projection against which to monitor early-warning indicators rather than a point prediction.

### 4.7 Comparison with related work

Briseno-Ramirez et al. (7) documented an approximately 5-year national dengue cycle (spectral peak 5.8 years) and a significant upward national trend (Sen’s slope approximately +1,275 cases/year) using STL decomposition, Butterworth filtering, and spectral analysis on the full 40-year national time series. Our state-level Mann–Kendall results are broadly consistent with their national trend finding. We extend their work by providing state-level spatial disaggregation, machine learning-based prospective forecasting, hazard covariate integration, and evaluation against published 2024–2025 ground truth. Martínez-Cadena et al. (8) proposed eco-epidemiological clustering with one-week-ahead Poisson boosting forecasts, achieving macro MAE = 17.1 and RMSE = 27.7, and found that autoregressive terms dominate over climate features, directly paralleling our SHAP finding that dengue_lag1 accounts for the dominant predictive share. Venegas-Ramírez et al. (27) analyzed 2022–2024 dengue incidence across 2,471 municipalities, identifying peak spatial autocorrelation (Moran’s I = 0.57) in 2023 and expansion of dengue-positive municipalities from 38 to 69% between 2022 and 2024. These complementary analyses collectively suggest that the next analytical priority for the Mexico dengue research community is a municipality-level forecasting framework integrating the temporal depth of the present study with the spatial granularity of Venegas-Ramírez et al. The reviewer-suggested benchmarks of recurrent neural network architectures (31–34) and data augmentation for rare epidemic years (35) represent methodological directions that are complementary to the present framework. Full LSTM or BiLSTM implementation is beyond the scope of this study’s core contributions: the EM-DAT hazard covariate framework and 41-year spatiotemporal characterization, but is explicitly identified as a priority for future iterations.

### 4.8 Limitations

Several study limitations are worth discussing. First, state-level aggregated data, even at the monthly level over 40 years, obscures within-state heterogeneity in dengue risk, particularly in large or ecologically diverse states such as Veracruz, Chiapas, and Sonora. Second, dengue serotype surveillance data at the monthly state level was not currently publicly accessible in Mexico through DGE/SINAVE or PAHO, which prevents integration as a time-varying model covariate; this is a data availability constraint rather than a methodological choice.; DENV-3 predominance from 2023 onward likely amplified 2024 severity beyond what climate and hazard features capture, and the immunological regime shift mechanism is discussed in Section 4.5. Third, the hazard covariate was aggregated nationally rather than at the state-level, due to EM-DAT geocoding metadata, particularly for events predating 2000. Notably, the spatial co-occurrence analysis (Section 3.4.4, Figure 5B) provides state-level evidence for geographic alignment between disaster hazard density and dengue incidence change, partially mitigating the national aggregation limitation for qualitative inference. Full state-level EM-DAT geocoding is a priority for future work. Fourth, the Severity Score is a researcher-derived composite index; sensitivity analysis confirmed robustness to weighting choices, but direct validation against state-level dengue outcomes would further strengthen the methodology. Fifth, covariate temporal coverage varies across sources and involves reconstruction over portions of the study period, as detailed in Section 2.3. Formal propagation of covariate reconstruction uncertainty into prediction intervals has not been implemented; this is identified as a future analytical priority, particularly for the 2026 forward projection. Sixth, the 2024 underestimate (−19.0%) and 2025 overestimate (+23.2%) reflect structural model boundary conditions discussed in Sections 4.5 and 4.6, respectively, Seventh, the 2026 forecast inherits uncertainty from climate climatology assumptions, autoregressive carry-forward of 2025 conditions, and the unmodeled trajectory of Mexico’s control strategy intensity. An explicit comparison against a parsimonious autoregressive time-series baseline (e.g., SARIMA) is identified as a future analytical priority that would further quantify the incremental ML contribution beyond temporal persistence (see Section 4.3).

### 4.9 Public health implications

This work has direct public health implications that warrant discussion. First, the geographic expansion of dengue into inland states documented by both the Mann–Kendall trend analysis and the 2024 Z-score anomalies necessitate expansion of dengue surveillance infrastructure, vector control investment, and clinical training beyond the traditional coastal endemic states. States with high Z-scores but currently low absolute burden (Zacatecas, Chihuahua, Querétaro) are precisely those least equipped for epidemic response—integrating data-driven model archetypes, like those derived here, into public health surveillance systems have pragmatic promise to deliver advance warning for real-time resource allocation to underequipped states. Second, the approximately 5-year epidemic cycle and the 2024 peak year suggest that 2029–2030 represents the next high-risk window, providing time for proactive surge capacity planning. Third, the hazard-adjusted forecasting framework has direct operational utility for post-disaster response: health authorities in storm- and flood-prone coastal states can use updated model projections in the weeks following major events, within the biologically plausible 0–2 month lag window, to pre-position resources before surveillance signals emerge. Finally, the analytical architecture developed here is directly transferable to similar ecologies, such as the southeastern United States, where dengue risk is expanding under climate change and is of particular relevance to military installations and tourism-dependent coastal economies facing emerging arboviral threats (36, 37).

## 5 Conclusion

This study presents the most comprehensive spatiotemporal analysis of dengue fever in Mexico to date, combining 41 years of state-level surveillance data with climate, land cover, and novel disaster severity covariates within a validated machine learning forecasting framework. The 2026 forward projection of 52,149–54,501 cases offer a baseline against which actual outcomes can be monitored, supporting early-warning surveillance during the inter-epidemic phase. Next, the inland geographic expansion of dengue, constitutes a shift in Mexico’s dengue epidemiology that demands a reorientation of surveillance and vector control investment toward previously unaffected regions. The 2025 epidemic decline following a record 2024 peak demonstrates that concerted public health action can interrupt dengue transmission even during a period of elevated environmental risk, and the model’s overestimate of 2025 provides a quantitative approximation of the cases averted by that intervention. Lastly, future work should extend this framework to the municipality level: incorporate serotype surveillance data as time-varying covariates; explore deep learning architectures (LSTM, BiLSTM) with data augmentation for improved handling of rare epidemic years (31–35); build state-level hazard aggregations from EM-DAT geocoding metadata, and develop a prospective real-time implementation for operational early warning in Mexico and transferable application to the southeastern United States and similar ecologies.
