# Supplementary material for: Geographic expansion, not viral intensification, leads to human dengue outbreaks in Mexico: a 40-year integrated remote sensing and machine learning analysis
Source: Front Public Health. 2026 Aug 3;14:1888753. doi: 10.3389/fpubh.2026.1888753 (PMC13478283; doi:10.3389/fpubh.2026.1888753)
Supplement: Supplementary file 1 [file Data_Sheet_1.DOCX]

**Supplemental Figure 1. Model Evaluation Dashboard*.***

*Panel A:* RMSE by model configuration on the 2022–2023 holdout (light = baseline; dark = hazard-adjusted; blue = Random Forest; red = XGBoost). *Panel B*: R² by model, with a dashed reference line at R² = 0.70. *Panel C:* MAE by model. *Panel D*: Predicted vs. actual cases on log–log scale for the XGBoost Hazard-Adjusted model on the 2022–2023 holdout, with the 1:1 line shown for reference. *Panel E*: Change in RMSE from adding hazard features (Hazard − Baseline) for each architecture; negative values indicate hazard-feature improvement. *Panel F*: National monthly forecast for 2024 (true out-of-sample), comparing observed cases (black) with the Ensemble Baseline (light blue) and Ensemble Hazard-Adjusted (red) predictions.
