# Supplementary material for: Geographic expansion, not viral intensification, leads to human dengue outbreaks in Mexico: a 40-year integrated remote sensing and machine learning analysis
Source: Front Public Health. 2026 Aug 3;14:1888753. doi: 10.3389/fpubh.2026.1888753 (PMC13478283; doi:10.3389/fpubh.2026.1888753)
Supplement: Supplementary file 2 [file Image_2.DOCX]

**Figure 2. SHAP and Feature Importance Analysis*.***

*Panel A:* Top-15 features ranked by mean |SHAP value|. Red bars indicate EM-DAT hazard features; dark blue bars indicate climate, temporal, or demographic features. *Panel B:* Proportional attribution of total absolute SHAP across thematic feature categories. Computed on a random sample of 400 observations from the 2022–2023 validation set using TreeExplainer.
