# Supplementary material for: Geographic expansion, not viral intensification, leads to human dengue outbreaks in Mexico: a 40-year integrated remote sensing and machine learning analysis
Source: Front Public Health. 2026 Aug 3;14:1888753. doi: 10.3389/fpubh.2026.1888753 (PMC13478283; doi:10.3389/fpubh.2026.1888753)
Supplement: Supplementary file 3 [file Supplementary_file_1.DOCX]

**Supplemental File 1: Equations used for variable calculations or spatial analysis**

**Equation 1**: Dengue age-standardized incidence rate per 100,000 population, per state per year

*IRₛ,ₜ* = (*Cₛ,ₜ* / *Pₛ,ₜ*) × 100,000

where *IRₛ,ₜ* is the incidence rate per 100,000 for state *s* at time *t*, *Cₛ,ₜ* is confirmed cases, and *Pₛ,ₜ* is the mid-year population estimate

**Equation 2:** Magnum’s formula for relative humidity

*RH* = 100 × $\frac{exp(\frac{a\cdot Td}{b + Td})}{exp(\frac{a\cdot T}{b + T})}$

where *T* is air temperature (°C), *Td* is dewpoint temperature (°C), *a* = 17.625, and *b* = 243.04°C.

**Equation 3**: Composite severity score

*S_e_* = $\sum_{d=1}^{10} w_{d}\cdot x_{e,d}$

where *S_e_* is the Severity Score for event *e*, *x{e,d}* ∈ {0,1} is the binary indicator for epidemiological impact dimension *d*, and $w_{d}$ = 1 for all *d* under equal weighting (sensitivity analyses tested $w_{d}$∈ {1, 2, 3})

**Equation 4**: Harmonic transformed seasonal encoding for climate covariates

*sin_monthₜ* = $sin(\frac{2\pi\cdot m_{t}}{12})$, *cos_month*ₜ = $cos(\frac{2\pi\cdot m_{t}}{12})$

where *m_t_* is the calendar month (1–12) at time *t*

**Equation 5:** Ensemble prediction formula combining random forest and XGBoost regression models

$ŷ_{s,t}^{Ensemble}$ = $\frac{1}{2}$($ŷ_{s,t}^{RF}+ ŷ_{s,t}^{XGB}$)

where $ŷ_{s,t}^{Ensemble}$ is the predicted dengue case count for state *s* in month *t*, and $ŷ_{s,t}^{RF}$and $ŷ_{s,t}^{XGB}$ are the Random Forest and XGBoost predictions, respectively

**Equation 6:** SHAP value decomposition conceptual equation

*f(x)* = $\phi_{0}$ + $\sum_{j=1}^{p} \phi_{j}$

where *f(x)* is the model prediction, $\phi_{0}$ is the baseline expected model output, $\phi_{j}$ is the SHAP value attributable to feature *j*, and *p* is the total number of features. SHAP values satisfy the efficiency axiom: $\sum_{j=1}^{p} \phi_{j}= f\left( x \right)- E[f(X)]$*.*

**Equation 7**: Mann-Kendal trend test

*S* = $\sum_{i=1}^{n-1} \sum_{j=j+1}^{n} sgn(x_{j}-x_{i}$)

Where n is the number of data points, $x_{j}$ is the data value at time *j* and $x_{i}$ is the data value at time *i,* and *sgn(*$x_{j}-x_{i})$ is the sign function as follows:

+1	if *θ* > 0

*sgn*(*θ*) =	0	if *θ* = 0

−1 if *θ* < 0.

**Equation 8**: Sen’s slope estimate formula

*β̂* = median ($\frac{x_{j}-x_{i}}{j-i}$) for all *i* < *j*

where *β̂* is Sen's slope estimator and additional variables are defined in equation 7, providing a robust estimate of the rate of change in incidence (cases per 100,000 per year)

**Equation 9:** Z-score anomaly for state-level dengue incidence rates

$Z_{s,j}$ = $\frac{{IR}_{s,j}- {ĪR}_{s}}{\sigma_{s}}$

Where *j* is 2024 or 2025,${ĪR}_{s}$ is the mean and $\sigma_{s}$is the standard deviation of annual incidence rates for state *s* over 1985–2023
