# Supplementary material for: Geographic expansion, not viral intensification, leads to human dengue outbreaks in Mexico: a 40-year integrated remote sensing and machine learning analysis
Source: Front Public Health. 2026 Aug 3;14:1888753. doi: 10.3389/fpubh.2026.1888753 (PMC13478283; doi:10.3389/fpubh.2026.1888753)
Supplement: Supplementary file 4 [file Table_1.DOCX]

**Supplemental Table 1. Holdout model performance (2022–2023 validation set, n = 768)**

| **Model** | **MAE** | **RMSE** | **R²** | **RMSE vs. Baseline** |
| --- | --- | --- | --- | --- |
| RF Baseline | 39.2 | 156.8 | 0.703 | — |
| RF Hazard-Adjusted | 39.9 | 156.9 | 0.702 | +0.1 (+0.1%) |
| XGBoost Baseline | 38.9 | 161.7 | 0.684 | — |
| XGBoost Hazard-Adjusted† | 39.5 | 160.5 | 0.688 | −1.2 (−0.7%) |

*† Best-performing model. Training: 1990–2021 (n = 12,032). MAE = mean absolute error (cases/month); RMSE = root mean squared error (cases/month). All metrics computed on the 2022–2023 temporal holdout set.*
