# Supplementary material for: Geographic expansion, not viral intensification, leads to human dengue outbreaks in Mexico: a 40-year integrated remote sensing and machine learning analysis
Source: Front Public Health. 2026 Aug 3;14:1888753. doi: 10.3389/fpubh.2026.1888753 (PMC13478283; doi:10.3389/fpubh.2026.1888753)
Supplement: Supplementary file 5 [file Table_2.DOCX]

**Supplemental Table 2.** Machine-learning models versus conventional temporal baselines, 2022–2023 holdout (n = 768 state-months; training 1990–2021).

| **Model** | **MAE** | **RMSE** | **R²** |
| --- | --- | --- | --- |
| RF Baseline | 39.2 | 156.8 | 0.703 |
| RF Hazard-Adjusted | 39.9 | 156.9 | 0.702 |
| XGBoost Baseline | 38.9 | 161.7 | 0.684 |
| XGBoost Hazard-Adjusted | 39.5 | 160.5 | 0.688 |
| SARIMA | 42.3 | 168.3 | 0.66 |
| SARIMAX (SARIMA + climate) | 43.1 | 168.7 | 0.66 |
| Naive persistence (lag-1) | 51.4 | 184.8 | 0.59 |
| Seasonal naive (lag-12) | 80.7 | 276.6 | 0.07 |

*Classical baselines evaluated one-step-ahead. The ML advantage over the seasonal-naive baseline is significant (Diebold–Mariano p < 0.01); over SARIMA and naive persistence it is directional but not significant on this holdout (p = 0.11 and 0.16). Baseline-versus-hazard differences are within sampling noise (state-cluster bootstrap 95% CI spans zero; Clark–West n.s.). MAE and RMSE in cases per month.*
